# Autoimmune pancreatitis: What we know so far

**DOI:** 10.1002/jgh3.12688

**Published:** 2021-12-10

**Authors:** Muaaz Masood

**Affiliations:** ^1^ Department of Internal Medicine Medical College of Georgia at Augusta University Augusta Georgia USA

**Keywords:** autoimmune pancreatitis, Type 1 autoimmune pancreatitis, Type 2 autoimmune pancreatitis

## Abstract

Autoimmune pancreatitis (AIP) is a rare, often‐missed disease that involves inflammation of the pancreas and strictures of the pancreatic duct. Its prevalence and incidence in the United States remain scarce. The disease has a varied presentation and often mimics pancreatic malignancy, which can make the diagnosis challenging. Most patients have an excellent response to corticosteroid therapy. Immunomodulators may be used in some cases. Rituximab is an effective, emerging treatment in steroid‐refractory cases. This study aims to review the two distinct types of AIP and provide a detailed analysis of the diagnostic approach and treatment modalities.

## Graphical abstract

Autoimmune pancreatitis is a rare, often‐missed disease that involves inflammation of the pancreas and strictures of the pancreatic duct. The disease has a varied presentation and often mimics pancreatic malignancy which can make the diagnosis challenging. This study aims to review the clinical manifestations of the two distinct types of autoimmune pancreatitis and provide a concise analysis of the diagnostic approach and latest treatment modalities

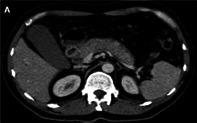

## Autoimmune pancreatitis

Autoimmune pancreatitis (AIP) has been seen mostly in Japanese case reports. Its prevalence, incidence, and reported cases in the United States, however, are largely unknown. The disease is chronic and manifests as acute attacks of pancreatitis with a quick response to steroids. The autoimmune aspect usually stems from high IgG levels and autoantibodies detected in the blood. The autoantibodies deposit in the pancreas and cause ductal strictures, narrowing, inflammation, and enlargement of the pancreas. AIP belongs to a large spectrum of IgG4‐related disease (IgG4‐RD) that share clinicopathological features and affect virtually every organ system. AIP may also co‐exist with other immunological dysfunctions.

### Pathophysiology

The pathogenesis of AIP is multifactorial involving an interplay of immunological, genetic, and environmental factors. AIP is associated with the infiltration of various immune cells into pancreatic tissue. The types of immune cells observed in Type 1 AIP include IgG4‐producing plasma cells and B‐lymphocyte antigen CD20. In Type 2 AIP, there is involvement of cluster of differentiation (CD) 4‐positive T cells and granulocytes.^1^ The pathogenesis of AIP has been associated with the cytotoxic T‐lymphocyte antigen 4 (CTLA‐4) gene, a negative regulator of T‐cell response.^2^ Single nucleotide polymorphisms (SNPs) involving the CTLA‐4 gene have been implicated in several autoimmune disorders such as Type 1 diabetes, autoimmune thyroid disease, autoimmune hepatitis, and primary biliary cirrhosis.^3^, ^4^, ^5^, ^6^ A soluble form of CTLA‐4 (sCTLA‐4) has also been shown to be increased in systemic lupus erythematosus, myasthenia gravis, and autoimmune thyroid disease.^7^ Umemura *et al*. concluded that AIP is associated with CTLA‐4 polymorphisms and is positively correlated with sCTLA‐4 levels.^8^

Serum IgG4 is elevated in patients with AIP and IgG4 antibodies characteristically deposit in affected organs, which results in fibrosis and obliterative phlebitis.^9^ IgG4 production is promoted by Th2 cells that produce IL‐10 and IL‐13 and regulatory T cells (Tregs), which produce IL‐10.^10^, ^11^ Studies have shown an enhanced T helper type 2 (Th2)‐mediated immune response in AIP.^12^, ^13^ Various additional cells types, including T follicular helper cells, CD4+ cytotoxic T cells, plasmacytoid dendritic cells, basophils, and monocytes upregulate IgG4 secretion and contribute to the pathogenetic mechanisms of AIP and IgG4‐RD.^10^

Eosinophils are thought to play a pathogenetic role in AIP. Peripheral eosinophilia and eosinophilic infiltrates have been observed in patients with AIP.^14^ There is also a high prevalence of allergic disorders in AIP based on a study by Kamisawa *et al*.^15^ Sah and colleagues reported a prevalence of 28% for peripheral eosinophilia and 15% for allergic disorders in patients with AIP.^14^ Interestingly, the Th2 immune response that is enhanced in AIP involves induction of IL‐4, IL‐5, and IL‐13, which leads to the expression of eotaxin‐3, a chemoattractant cytokine for eosinophils to be directed to inflammatory sites, via the STAT6 pathway.^16^ Mari *et al*. suggested that Th2 cytokine‐induced eotaxin‐3 expression plays a role in the pathophysiology of pancreatic disorders such as AIP and eosinophilic pancreatitis.^16^

Recently, interferon‐I (IFN‐I) has been linked to the immunopathogenesis of AIP. AIP has been linked to increased levels of IFN‐I produced by plasmacytoid dendritic cells.^17^ IFN‐I is responsible for increased IL‐33, which is involved in the induction of the fibroinflammatory process in the pancreatic duct cells.^18^ IFN‐I also stimulates plasma cells to produce IgG4.^19^ The dysregulation of the IFN‐I system has also been implicated in several autoimmune rheumatic disorders such as systemic lupus erythematosus, rheumatoid arthritis, Sjogren's syndrome, and inflammatory myositis.^20^

Environmental causes of AIP have also been explored in the setting of antigen exposure and its effect on serum IgG4 concentration. IgG4 levels have been reported to be upregulated with chronic immune stimulation as evidenced by elevated bee‐venom‐specific IgG4 levels in an analysis of beekeepers by Garcia‐Robaina *et al*.^21^ Wenninger *et al*. suggested that chronic exposure to occupational antigens, that is, solvents, industrial or metal dust, pigments, and oils may also be associated with the initiation or maintenance of IgG4‐RD.^22^

Patients with AIP also have high levels of multiple, nonspecific antibodies, including antinuclear antibodies, anticarbonic anhydrase II antibodies, pancreatic secretory trypsin inhibitor antibodies, and antilactoferrin antibodies.^18^ The role of IgG4 antibodies in the pathogenesis of AIP remains unknown.^10^ IgG4 is highly versatile and regulates immune responses in both beneficial and detrimental ways. On one hand, IgG4 plays a protective role in hypersensitivity reactions and allergen‐specific immunotherapy.^21^, ^22^ On the other hand, IgG4 has been shown to be pathogenic in autoimmune diseases such as pemphigus. IgG4 is also produced by helminths in the presence of parasitic diseases and by tumor cells in malignancies such as melanoma and cholangiocarcinoma.^9^ In a study by Shiokawa that examined the pathogenicity of IgG in patients with IgG4‐RD by injecting the IgGs into neonatal mice, pancreatic and salivary gland injuries were noted after injection of IgG, with more destructive changes induced by IgG1 compared to IgG4.^23^ Interestingly, the deleterious effect of IgG1 was inhibited by simultaneous injection of IgG4. This data suggest that IgG4 may have a possible protective or attenuating role in IgG4‐RD, including AIP. Further studies are needed to elucidate the role of IgG4 in the pathogenesis of AIP.

### Epidemiology

The overall prevalence and incidence of AIP are largely unknown. Japanese data have reported the prevalence as 4.6 per 100 000 population and the incidence as 1.4 per 100 000.^24^ AIP comprises 5–6% of all cases of chronic pancreatitis.^25^ There is an association of AIP with human leukocyte antigen (HLA) serotypes DRB1*0405 and DQB1*0401.^26^ AIP has increasingly been reported in Western countries and is thus a worldwide entity.

### Clinical manifestations

The clinical manifestations of AIP are varied thus making it challenging to diagnose AIP based on symptomatology alone. Painless obstructive jaundice is the most common presentation.^27^ Jaundice in AIP is likely related to the involvement of the biliary tract, the most common extrapancreatic manifestation, affecting up to 65.9% of patients with AIP based on a review by Meng *et al*.^28^ Other less common symptoms include mild abdominal or back pain, fatigue, weight loss, pancreatic mass, or chronic pancreatitis.^27^, ^29^ Abdominal pain in Type 1 AIP may be mild to none and does not classically resemble the severity observed in acute pancreatitis.^30^ In contrast, 68% of patients with Type 2 AIP commonly present with acute, painful pancreatitis.^27^ Some patients with AIP are clinically asymptomatic. AIP has been associated with pancreatic exocrine dysfunction in approximately 80% of patients and endocrine dysfunction, that is, diabetes mellitus in 70% of cases.^31^ Patients may report symptoms of polydipsia and polyuria. Diabetes mellitus can occur before, simultaneously, or after steroid treatment.^32^ Patients may also present with a variety of manifestations due to other organ involvement, including but not limited to sclerosing cholangitis, Sjogren's syndrome, orbital pseudotumor, lung nodules, hypophysitis, thyroiditis, prostatitis, interstitial nephritis, and retroperitoneal fibrosis.^33^ Studies aimed to analyze the clinical characteristics and treatment modalities for AIP from different countries have had varying results. A study from China observed jaundice in 72% and abdominal pain in 44% of AIP patients whereas a multicenter study based in Spain reported abdominal pain in 65.4% and jaundice in 51.9% of AIP patients.^34^, ^35^ Hardacre *et al*. reported jaundice in 84% and abdominal pain in 54% of patients with AIP.^36^ Weber and colleagues observed jaundice and abdominal pain in 68% and 55% of AIP patients, respectively.^37^

AIP presenting as a focal pancreatic mass or enlargement can often mimic pancreatic malignancy. There is also clinical overlap between AIP and pancreatic cancer with symptoms of obstructive jaundice, weight loss, and mild abdominal pain shared by both diseases. It is, therefore, very important to exclude malignancy in these patients.^38^ A review reported that 29.7% of the patients were misdiagnosed with pancreatic cancer and these patients underwent surgical intervention.^28^ A systematic approach should be undertaken in distinguishing AIP from pancreatic cancer. Obstructive jaundice in pancreatic cancer tends to be progressive in nature whereas jaundice of AIP may fluctuate or spontaneously improve.^39^ Marked cachexia, inability to tolerate oral intake, or pain requiring narcotics are more suggestive of pancreatic cancer than AIP.^29^ Serum IgG4 levels may be elevated in 10% of patients with pancreatic cancer but only 1% of patients had IgG4 levels >280 mg/dL.^40^ Presence of other organ involvement is suggestive of AIP over pancreatic cancer.^41^ It is important to note that pancreatic cancer is far more common than AIP. Patients without characteristic features of either pancreatic cancer or AIP should be evaluated for pancreatic malignancy first.^29^ The radiologic differences between AIP and pancreatic cancer are discussed with imaging modalities below.

There are two major classifications of AIP that are defined by unique features (Table 1). Type 1 AIP, also known as lymphoplasmacytic sclerosing pancreatitis (LPSP), typically present in late adulthood with a mean age of diagnosis of 50 years and older and affects males three times more commonly than females.^42^ Type 1 AIP may be a manifestation of a spectrum of IgG4‐RD characterized by widespread, multi‐organ involvement, which may include the eyes (pseudolymphoma), bile ducts (sclerosing cholangitis), lymph nodes (mediastinal/intraabdominal/hilar adenopathy), salivary glands (sclerosing sialadenitis), thyroid (Riedel's thyroiditis), kidneys (interstitial nephritis), and lungs (nodules, mediastinal fibrosis).^30^, ^42^, ^43^ The diagnosis can usually be made clinically and histology is not necessary to diagnose Type 1 AIP.^29^ New‐onset diabetes mellitus and abnormal pancreatic exocrine function may occur.^44^ Type 1 AIP may be allergic in origin.^15^

**Table 1:** Comparison of key characteristics of the two distinct types of autoimmune pancreatitis

	Type 1 autoimmune pancreatitis	Type 2 autoimmune pancreatitis
Age of onset	>50 years old	30–50 years old
Gender	Male predilection	Equally affects males and females
Geographical distribution	More common in Asia	More common in the United States and Europe
Histological characteristics	Lymphoplasmacytic sclerosing pancreatitis; absent granulocytic epithelial lesions	Idiopathic duct‐centric pancreatitis; presence of granulocytic epithelial lesions
Serum IgG4 level	Elevated	Normal
Extra‐pancreatic manifestations	Multi‐organ involvement (biliary tract, retroperitoneum, renal, salivary gland, lung)	None
Association with inflammatory bowel disease	Rare	Common
Diagnosis	May be established clinically	Pancreatic biopsy required
Steroid responsiveness	High	High
Relapse rates	High	Low

Type 2 AIP, also known as idiopathic duct‐centric pancreatitis (IDCP), affects males and females equally and has a younger mean age of diagnosis (43 years) compared to Type 1 AIP. Type 2 AIP lacks systemic involvement or IgG4 elevation.^45^ Approximately, 30% of cases of Type 2 AIP are associated with inflammatory bowel disease with a predilection for ulcerative colitis compared to Crohn's disease.^46^ A study by Sah and colleagues reported that ulcerative colitis was present in 15.8% of patients with Type 2 AIP.^47^ Histology for Type 2 AIP typically shows duct‐centric pancreatitis with granulocytic epithelial lesions, which can eventually obliterate the pancreatic duct.^48^ Diagnosis requires a pancreatic biopsy. Type 2 AIP has lower relapse rates compared to Type 1 AIP.^47^

### Diagnosis

There are two widely utilized diagnostic criteria for AIP. The Japanese guidelines developed by Okazaki *et al*. include three components—namely imaging, serology, and histology.^31^ The Mayo Clinic HISORt criteria consist of five components, including **H**istology, **I**maging, **S**erology, **O**rgan involvement, and **R**esponse to steroid **t**herapy.^49^ The International Consensus Diagnostic Criteria, developed in 2011 after the review of existing criteria, divided each of the five components into levels of evidence: typical/highly suggestive of AIP (Level 1) and indeterminate/suggestive of AIP (Level 2).^29^ Recently, Chari *et al*. proposed a revision to the HISORt diagnostic criteria for AIP, which stratified patients into three groups: (i) highly likely to have AIP, (ii) highly likely to have pancreatic cancer, and (ii) indeterminate based on key imaging findings.^41^

Histological features of Type 1 AIP include lymphoplasmacytic infiltration of tissues with greater than 10 IgG4‐positive cells per high‐power field, storiform fibrosis (swirling pattern), and obliterative phlebitis.^45^ Characteristic histological findings of Type 2 AIP are granulocytic epithelial lesions, which involve infiltration of neutrophils in the lumen and epithelium of pancreatic ducts/acini that eventually cause obliteration of duct lumen.^50^ There are minimal IgG4‐positive cells present in Type 2 AIP.^29^

**Figure 1:**
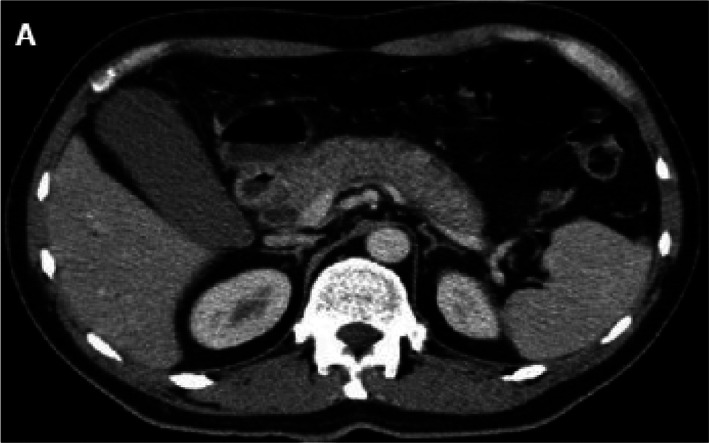
Contrast‐enhanced CT reveals diffuse pancreatic enlargement (i.e. sausage pancreas) with “featureless borders” and a capsule‐like rim, which is characteristic of AIP.^46^

The initial imaging modalities of choice to evaluate patients for suspected AIP are contrast‐enhanced computed tomography (CT) scan and magnetic resonance imaging (MRI). CT may reveal diffuse pancreatic enlargement (i.e. sausage pancreas) with “featureless borders” and delayed enhancement, which is characteristic of AIP.^48^ A capsule‐like rim is also a significant CT finding and may result from the presence of inflammatory cells and fibrosis^51^ (Fig. 1). However, some patients may have a normal pancreas.^27^ In comparison, CT features suggestive of pancreatic cancer include low‐density mass, pancreatic ductal dilation/cutoff with or without pancreatic atrophy.^29^, ^41^ MRI for AIP may demonstrate a hypointense T1 signal in the setting of fibrosis and a hypointense capsule‐like rim on T1 and T2‐weighted images.^52^

Magnetic resonance cholangiopancreatography (MRCP) may be obtained for further characterization using a noninvasive method and may show narrowing of the main pancreatic duct as well as the common bile duct.^45^ MRCP is less accurate than endoscopic retrograde cholangiopancreatography (ERCP) in distinguishing focal AIP from pancreatic cancer.^53^, ^54^ ERCP can aid in the diagnosis of AIP and allow for the intervention of ductal obstruction as warranted. ERCP is a generally safe procedure and the incidence of ERCP‐related adverse events in patients with Type 1 AIP is reported to be low.^55^ Studies have defined four important diagnostic features for AIP on MRCP/ERCP: long stricture (>1/3 the length of the pancreatic duct), lack of upstream dilatation from the stricture (<5 mm), multiple strictures, and side branches arising from a segment with stricture.^56^, ^57^, ^58^ Diffuse or segmental irregular narrowing of the main pancreatic duct is characteristic of AIP.^59^ The narrowing of the pancreatic duct lumen is likely due to compression by lymphoplasmacytic infiltration and fibrosis.^60^ The presence of migrating strictures in AIP is uncommon in most other disorders of pancreatic or biliary systems.^61^ A positive duct‐penetrating sign (a nonobstructed, visible main pancreatic duct and/or common bile duct lumen penetrating a pancreatic mass) and icicle sign (progressive decrease in the diameter of the main pancreatic duct) can be useful in diagnosing and distinguishing AIP from pancreatic cancer.^62^, ^63^ It is important to note that ERCP alone is limited in diagnosing AIP and should be used in conjunction with clinical, serological, and histological data.^56^ ERCP is also not a reliable diagnostic modality to distinguish IgG4‐associated cholangitis from primary sclerosing cholangitis and cholangiocarcinoma.^64^

Endoscopic ultrasound (EUS) can be used for the evaluation of pancreatic parenchyma and the pancreaticobiliary ducts. EUS may show a hypoechogenic pancreatic enlargement in over 50% of AIP patients and hypoechogenic foci located irregularly in the pancreas in about 40% of AIP patients.^65^ EUS may also reveal glandular atrophy, calcifications, cystic spaces, or a normal pancreas.^66^ These features are not specific to AIP and may be seen in other pancreatic disorders. Given the lack of specific diagnostic features identified and the heterogeneity among the findings, the utility of EUS alone in diagnosing AIP is limited. One advantage of EUS is the ability to obtain tissue using a EUS‐guided fine needle aspiration or core biopsy/resection to establish a histological diagnosis and to rule out pancreatic cancer. However, the diagnosis of AIP with EUS‐guided fine needle aspiration (FNA) is often challenging due to the small sample size.^67^ EUS‐guided FNA alone has a low sensitivity (up to 40%) but has increased sensitivity (up to 85%) when combined with Trucut biopsy.^68^, ^69^ A multicenter study of 78 patients evaluated the use of the 22‐gauge needle in EUS‐FNA. Pancreatic tissue with at least one high‐powered field (HPF) was obtained from 80% of patients and 60% of patients were diagnosed with ICDC Level 2 or higher.^70^ A study from Japan involving 110 patients with suspected AIP also concluded that the 22‐gauge needle obtained a significantly greater number of HPFs compared to the 20‐gauge needle.^71^ In the presence of a pancreatic head mass consistent with AIP on imaging, the ICDC guidelines recommend a EUS‐guided core biopsy or resection.^29^, ^72^ Biopsy revealing positive IgG4 immunostaining from the major duodenal papilla supports the diagnosis of AIP.^73^ Of note, EUS may assist with differentiating AIP from cholangiocarcinoma. Bile ducts in AIP may reveal concentric wall thickening with a smooth luminal surface.^74^ In comparison, bile ducts in cholangiocarcinoma characteristically have eccentric wall thickening and an irregular luminal surface.^33^

Serum IgG4 is a useful diagnostic test in patients for whom AIP is suspected. IgG4 accounts for 5–6% of total serum IgG in the healthy population.^75^ Normal serum IgG4 is typically <140 mg/dL and values ≥280 mg/dL, or twice the upper limit of normal, have been shown to be highly specific to AIP.^41^ Serum IgG4 levels >140 mg/dL have also been reported to be diagnostic of AIP with 86% sensitivity and 90–96% specificity.^45^ It is important to note that elevations in serum IgG4 can occur in 10% of patients with pancreatic cancer.^40^, ^76^ Elevated IgG4 has also been reported in allergic conditions, parasitic infections, and pemphigus vulgaris.^77^, ^78^, ^79^ However, elevations in IgG4 in these conditions are often less than twice the upper limit of normal, which led to ≥280 mg/dL as the most specific cutoff value for AIP.^40^ Mild elevations between 135 and 200 mg/dL should be interpreted cautiously and may warrant further evaluation.^76^

### Treatment

The response to steroid therapy is one of the HISORt diagnostic criteria for AIP.^49^ Overall, there has been a lack of randomized, controlled clinical trials to guide treatment decisions in AIP. Retrospective data suggest that corticosteroids have been shown to be effective in inducing remission, reducing symptoms and improving inflammation, strictures, and pancreatic enlargement.^80^, ^81^, ^82^ The majority of patients with AIP respond to steroid therapy although patients with biliary strictures tend to have a more variable response rate.^83^ The usual treatment regimen includes prednisone 40 mg/day or prednisolone 0.6–1 mg/kg/day for 4 weeks' duration, followed by a gradual taper of 5 mg/week.^49^ Response can be assessed clinically with improvement in symptoms and objectively by serum IgG4 levels, liver function tests, and repeat imaging during or after the 4‐ to 6‐week course of treatment.^68^, ^84^ For patients undergoing a diagnostic steroid trial, repeat imaging is recommended sooner (at 2 weeks) and is expected to show improvement.^46^, ^82^ A lack of improvement or response with steroids may be a sign of an alternate diagnosis such as pancreatic malignancy. The confirmation of histological remission is not recommended due to the challenges of performing biopsies.^85^ Endoscopic biliary stent placement may be used to relieve obstructive jaundice, which can be removed 6–8 weeks after the initiation of steroid therapy.^85^

Following induction with steroid therapy, the decision to initiate maintenance therapy should be individualized based on the patient's risk for disease relapse, morbidity from disease relapse, and the adverse effects of maintenance therapy. Patients with Type 1 AIP are at the highest risk for disease relapse. There is a low relapse risk with Type 2 AIP and thus steroid therapy is typically tapered off and discontinued following induction in these patients.^86^ Based on the International Association of Pancreatology guidelines, patients at increased risk for disease relapse include those with proximal biliary disease, diffuse pancreatic enlargement, persistently elevated IgG4 levels following steroid induction, delayed radiographic remission, and disease involving two or more organs.^87^ Elevated serum baseline IgG4, IgE, and eosinophilia predicted relapse in IgG4‐RD.^88^ Recent guidelines from Japan suggest low‐dose steroids (i.e. 2.5–5 mg) as maintenance therapy, which may be stopped in 6–12 months.^89^ A multicenter study of 563 patients with AIP in Japan concluded that patients on maintenance therapy with low‐dose steroids had lower relapse rates compared to those who discontinued maintenance therapy, though the treatment regimens used were highly variable.^90^ A randomized controlled trial by Masamune *et al*. demonstrated that maintenance therapy with oral prednisolone 5–7.5 mg/day for 3 years reduced disease relapses compared to those that discontinued steroids after 26 weeks.^91^ The trial had limitations, including a small sample size due to the inability to extend the participant recruitment period and unbalanced, unmasked allocation between treatment groups. Despite this, the study is the first randomized controlled trial to suggest a beneficial effect of steroids when used as maintenance therapy in patients with AIP. Further studies are warranted to investigate the efficacy of steroid maintenance therapy regimens. Other experts suggest close monitoring and as‐needed corticosteroids to minimize adverse effects.^33^ An alternative approach is the use of steroid‐sparing immunomodulators, that is, azathioprine, 6‐mercaptopurine (6‐MP), or mycophenolate mofetil (MMF). The optimal dose and duration for immunomodulator maintenance therapy have not been defined. Azathioprine, 6‐MP, and MMF have similar efficacy and can be substituted.^92^ Azathioprine at doses of 2–2.5 mg/kg (used in inflammatory bowel disease) is more effective than 1 mg/kg or a fixed dose of 50 mg (used in autoimmune hepatitis). Ultimately, the decision for a maintenance therapy agent should be individualized based on the patient's disease severity, patient preference, treatment adherence, and adverse effects of treatment.

The use of empiric steroids as an approach to diagnose AIP should generally be avoided in the absence of other evidence (i.e. histology, imaging, serum markers, and extrapancreatic manifestations) to suggest AIP. Steroids may exert a placebo effect in patients with pancreatic malignancy, which could be mistaken for steroid response.^29^ In special cases where there remains the need to differentiate AIP and pancreatic cancer after a thorough, nondiagnostic investigation, a steroid trial may be considered but it should be performed with caution and under the guidance of pancreatic specialists.^82^ Regarding the course of diabetes associated with AIP, approximately half of the patients with AIP were observed to have an improvement in diabetes after steroid therapy.^32^ About 20% of patients developed new‐onset diabetes or worsened glycemic control following steroid therapy with a higher incidence of these findings noted in elderly patients.^32^

Relapses may occur in 53% of patients and are more common in Type 1 AIP compared to Type 2 AIP (<10%).^47^ Disease relapse involves abnormalities in laboratory or imaging studies. Relapses may affect the pancreas or occur in another previously unaffected organ within the spectrum of IgG4‐RD.^93^ There is a lack of consensus on the definition of disease relapse in AIP. However, a clinical symptom alone (i.e. abdominal pain without evidence of pancreatic inflammation) or an isolated serum IgG4 elevation (without supporting radiologic or biochemical findings) can occur independent of disease activity, and therefore, may not represent relapsed disease.^94^ Re‐induction with steroids has shown to be very effective with high remission rates achieved.^94^, ^95^ Overall, there remains a lack of robust data to guide the management of relapsed disease in AIP. One treatment option includes a high‐dose corticosteroid regimen for 4–6 weeks followed by a gradual steroid taper of 5 mg/week and either maintenance steroids or steroid discontinuation.^96^ For steroid‐refractory cases, immunomodulators (i.e. azathioprine) or single‐agent rituximab, a monoclonal antibody against CD20 antigen on B cells, have been used. Immunomodulators are not effective as monotherapy and typically require an overlap with steroids.^92^ A treatment regimen involving high‐dose corticosteroids in combination with azathioprine followed by steroid taper and discontinuation has been proposed.^86^ Rituximab can be utilized for both induction and maintenance therapy and is the only choice for patients who are intolerant or resistant to steroids and immunomodulators. Induction involves either 4 weekly doses of 375 mg/m^2^ body surface area (BSA) or 2 doses of 1000 mg each administered 2 weeks apart.^92^, ^97^ Rituximab has shown a higher efficacy rate than immunomodulators for maintenance therapy in a study based in France though it is often costly.^98^ A suggested treatment plan includes rituximab induction therapy followed by rituximab maintenance therapy 375 mg/m^2^ BSA every 2–3 months for 2 years.^92^ Clinical response is high with rituximab (>90% in patients with IgG4‐RD) and disease relapse and adverse effects are low.^88^ Induction therapy without maintenance therapy may lead to a higher relapse rate.^99^

The long‐term prognosis of AIP is not yet unknown. Many patients with AIP were discovered to have malignancies either at the time of AIP diagnosis or within one year.^100^, ^101^ The three most commonly diagnosed conditions were gastric, colorectal, and bladder cancer.^102^ The underlying mechanisms are not known. It is hypothesized that there may be a component of paraneoplastic syndrome associated with AIP.^103^ Moreover, a small study of 63 patients suggested that patients with Type 1 AIP have an elevated risk for pancreatic cancer similar to patients with chronic pancreatitis.^104^ Further studies are needed to define the risk and relationship between AIP and malignancy. The effect of AIP on mortality also remains an area yet to be explored.^85^

## Conclusion

AIP is a rare, often‐missed disease that involves inflammation of the pancreas and strictures of the pancreatic duct. The immunologic component of pancreatitis should be considered in patients presenting with pancreatitis without a significant history of alcohol, drugs, or gallstones. AIP can often mimic pancreatic malignancy and it is, therefore, very important to exclude pancreatic malignancy in these patients. Most patients have an excellent response to corticosteroid therapy. Immunomodulators may be used in some cases. Rituximab is an effective, emerging treatment in steroid‐refractory cases. Further studies are warranted to determine optimal dosing/duration of medical therapy and the prevention of relapse.

## References

[1] Pezzilli R , Pagano N . Pathophysiology of autoimmune pancreatitis. World J. Gastrointest. Pathophysiol. 2014; 5(1): 11–7. 24891971 10.4291/wjgp.v5.i1.11 PMC4024516

[2] Chang MC , Chang YT , Tien YW *et al*. T‐cell regulatory gene CTLA‐4 polymorphism/haplotype association with autoimmune pancreatitis. Clin. Chem. 2007; 53(9): 1700–5. 17712006 10.1373/clinchem.2007.085951

[3] Yang X‐C , Fujino M , Cai S‐J *et al*. Genetic polymorphisms of cytotoxic T‐lymphocyte antigen 4 in primary biliary cholangitis: a meta‐analysis. J. Immunol. Res. 2017; 2017: 5295164. 28642883 10.1155/2017/5295164 PMC5470032

[4] Kavvoura FK , Ioannidis JPA . CTLA‐4 gene polymorphisms and susceptibility to Type 1 diabetes mellitus: A HuGE review and meta‐analysis. Am. J. Epidemiol. 2005; 162(1): 3–16. 15961581 10.1093/aje/kwi165

[5] Agarwal K , Czaja AJ , Jones DE *et al*. Cytotoxic T lymphocyte antigen‐4 (CTLA‐4) gene polymorphisms and susceptibility to type 1 autoimmune hepatitis. Hepatology. 2000; 31(1): 49–53. 10613727 10.1002/hep.510310110

[6] Chistiakov DA , Turakulov RI . CTLA‐4 and its role in autoimmune thyroid disease. J. Mol. Endocrinol. 2003; 31(1): 21–36. 12914522 10.1677/jme.0.0310021

[7] Simone R , Pesce G , Antola P *et al*. The soluble form of CTLA‐4 from serum of patients with autoimmune diseases regulates T‐cell responses. Biomed. Res. Int. 2014; 2014: 215763. 24605322 10.1155/2014/215763 PMC3925529

[8] Umemura T , Ota M , Hamano H *et al*. Association of autoimmune pancreatitis with cytotoxic T‐lymphocyte antigen 4 gene polymorphisms in Japanese patients. Am. J. Gastroenterol. 2008; 103(3): 588–94. 18341485 10.1111/j.1572-0241.2007.01750.x

[9] Trampert DC , Hubers LM , van de Graaf SFJ *et al*. On the role of IgG4 in inflammatory conditions: lessons for IgG4‐related disease. Biochim. Biophys. Acta Mol. Basis Dis. 2018; 1864(4 Pt B): 1401–9. 28782655 10.1016/j.bbadis.2017.07.038

[10] Liu C , Zhang P , Zhang W . Immunological mechanism of IgG4‐related disease. J. Transl. Autoimmun. 2020; 3: 100047. 32743528 10.1016/j.jtauto.2020.100047 PMC7388377

[11] Miyoshi H , Uchida K , Taniguchi T *et al*. Circulating naïve and CD4+CD25high regulatory T Cells in patients with autoimmune pancreatitis. Pancreas. 2008; 36(2): 133–40. 18376303 10.1097/MPA.0b013e3181577553

[12] Zen Y , Fujii T , Harada K *et al*. Th2 and regulatory immune reactions are increased in immunoglobin G4‐related sclerosing pancreatitis and cholangitis. Hepatology. 2007; 45(6): 1538–46. 17518371 10.1002/hep.21697

[13] Zhou Q , Tao X , Xia S *et al*. T lymphocytes: a promising immunotherapeutic target for pancreatitis and pancreatic cancer? Frontiers. Oncology. 2020; 10(382). 10.3389/fonc.2020.00382 PMC7105736 32266154

[14] Sah RP , Pannala R , Zhang L *et al*. Eosinophilia and allergic disorders in autoimmune pancreatitis. Off. J. Am. Coll. Gastroenterol. | ACG. 2010; 105(11): 2485–91. 10.1038/ajg.2010.236 20551940

[15] Kamisawa T , Anjiki H , Egawa N *et al*. Allergic manifestations in autoimmune pancreatitis. Eur. J. Gastroenterol. Hepatol. 2009; 21(10): 1136–9. 19757521 10.1097/meg.0b013e3283297417

[16] Mari A , Kadah A , Mahamid M *et al*. IgG4 related autoimmune pancreatitis: an overview and the emerging role of serum eotaxin as a potential treatment target. Isr. Med. Assoc. J. 2019; 21(9): 620–3. 31542909

[17] Arai Y , Yamashita K , Kuriyama K *et al*. Plasmacytoid dendritic cell activation and IFN‐α production are prominent features of murine autoimmune pancreatitis and human IgG4‐related autoimmune pancreatitis. J. Immunol. 2015; 195(7): 3033–44. 26297761 10.4049/jimmunol.1500971

[18] Blaho M , Dítě P , Kunovský L *et al*. Autoimmune pancreatitis – An ongoing challenge. Adv. Med. Sci. 2020; 65(2): 403–8. 32805624 10.1016/j.advms.2020.07.002

[19] Minaga K , Watanabe T , Hara A *et al*. Plasmacytoid dendritic cells as a new therapeutic target for autoimmune pancreatitis and IgG4‐related disease. Front. Immunol. 2021; 12: 713779. 34367181 10.3389/fimmu.2021.713779 PMC8342887

[20] Psarras A , Emery P , Vital EM . Type I interferon–mediated autoimmune diseases: pathogenesis, diagnosis and targeted therapy. Rheumatology. 2017; 56(10): 1662–75. 28122959 10.1093/rheumatology/kew431

[21] García‐Robaina JC , De La Torre‐Morín F , Vazquez‐Moncholi C *et al*. The natural history of Apis‐specific IgG and IgG4 in beekeepers. Clin. Exp. Allergy. 1997; 27(4): 418–23. 9146935

[22] de Buy Wenniger LJM , Culver EL , Beuers U . Exposure to occupational antigens might predispose to IgG4‐related disease. Hepatology. 2014; 60(4): 1453–4. 24407836 10.1002/hep.26999 PMC4258085

[23] Shiokawa M , Kodama Y , Kuriyama K *et al*. Pathogenicity of IgG in patients with IgG4‐related disease. Gut. 2016; 65(8): 1322–32. 26964842 10.1136/gutjnl-2015-310336

[24] Kamisawa T , Shimosegawa T . Epidemiology of autoimmune pancreatitis. Pancreas. 2018: 503–9.

[25] Nishimori I , Tamakoshi A , Otsuki M . Prevalence of autoimmune pancreatitis in Japan from a nationwide survey in 2002. J. Gastroenterol. 2007; 42(Suppl 18): 6–8. 17520216 10.1007/s00535-007-2043-y

[26] Kawa S , Ota M , Yoshizawa K *et al*. HLA DRB10405‐DQB10401 haplotype is associated with autoimmune pancreatitis in the Japanese population. Gastroenterology. 2002; 122(5): 1264–9. 11984513 10.1053/gast.2002.33022

[27] Omiyale AO . Autoimmune pancreatitis. Gland Surg. 2016; 5(3): 318–26. 27294040 10.21037/gs.2015.11.02 PMC4884700

[28] Meng Q , Xin L , Liu W *et al*. Diagnosis and treatment of autoimmune pancreatitis in China: A systematic review. PLoS One. 2015; 10(6): e0130466. 26110658 10.1371/journal.pone.0130466 PMC4481503

[29] Shimosegawa T , Chari ST , Frulloni L *et al*. International consensus diagnostic criteria for autoimmune pancreatitis: guidelines of the International Association of Pancreatology. Pancreas. 2011; 40(3): 352–8. 21412117 10.1097/MPA.0b013e3182142fd2

[30] Kim E , Voaklander R , Kasmin FE *et al*. Autoimmune pancreatitis: a multiorgan disease presenting a conundrum for clinicians in the West. Gastroenterol. Hepatol. (NY). 2015; 11(9): 606–11. PMC4965620 27482182

[31] Okazaki K , Kawa S , Kamisawa T *et al*. Japanese clinical guidelines for autoimmune pancreatitis. Pancreas. 2009; 38(8): 849–66. 19745774 10.1097/MPA.0b013e3181b9ee1c

[32] Nishimori I , Tamakoshi A , Kawa S *et al*. Influence of steroid therapy on the course of diabetes mellitus in patients with autoimmune pancreatitis: findings from a nationwide survey in Japan. Pancreas. 2006; 32(3): 244–8. 16628078 10.1097/01.mpa.0000202950.02988.07

[33] Ketwaroo GA , Sheth S . Autoimmune pancreatitis. Gastroenterol. Rep. (Oxf.). 2013; 1(1): 27–32. 24759664 10.1093/gastro/got011 PMC3768278

[34] Song Y , Liu QD , Zhou NX *et al*. Diagnosis and management of autoimmune pancreatitis: experience from China. World J. Gastroenterol. 2008; 14(4): 601–6. 18203294 10.3748/wjg.14.601 PMC2681153

[35] López‐Serrano A , Crespo J , Pascual I *et al*. Diagnosis, treatment and long‐term outcomes of autoimmune pancreatitis in Spain based on the International Consensus Diagnostic Criteria: A multi‐centre study. Pancreatology. 2016; 16(3): 382–90. 26944001 10.1016/j.pan.2016.02.006

[36] Hardacre JM , Iacobuzio‐Donahue CA , Sohn TA *et al*. Results of pancreaticoduodenectomy for lymphoplasmacytic sclerosing pancreatitis. Ann. Surg. 2003; 237(6): 853–9. 12796582 10.1097/01.SLA.0000071516.54864.C1 PMC1514684

[37] Weber SM , Cubukcu‐Dimopulo O , Palesty JA *et al*. Lymphoplasmacytic sclerosing pancreatitis: inflammatory mimic of pancreatic carcinoma. J. Gastrointest. Surg. 2003; 7(1): 129–37 discussion 37–9. 12559194 10.1016/s1091-255x(02)00148-8

[38] Kamisawa T , Egawa N , Nakajima H *et al*. Clinical difficulties in the differentiation of autoimmune pancreatitis and pancreatic carcinoma. Am. J. Gastroenterol. 2003; 98(12): 2694–9. 14687819 10.1111/j.1572-0241.2003.08775.x

[39] Takuma K , Kamisawa T , Gopalakrishna R *et al*. Strategy to differentiate autoimmune pancreatitis from pancreas cancer. World J. Gastroenterol. 2012; 18(10): 1015–20. 22416175 10.3748/wjg.v18.i10.1015 PMC3296974

[40] Ghazale A , Chari ST , Smyrk TC *et al*. Value of serum IgG4 in the diagnosis of autoimmune pancreatitis and in distinguishing it from pancreatic cancer. Am. J. Gastroenterol. 2007; 102(8): 1646–53. 17555461 10.1111/j.1572-0241.2007.01264.x

[41] Chari ST , Takahashi N , Levy MJ *et al*. A diagnostic strategy to distinguish autoimmune pancreatitis from pancreatic cancer. Clin. Gastroenterol. Hepatol. 2009; 7(10): 1097–103. 19410017 10.1016/j.cgh.2009.04.020

[42] Chari ST , Longnecker DS , Klöppel G . The diagnosis of autoimmune pancreatitis: a Western perspective. Pancreas. 2009; 38(8): 846–8. 19855232 10.1097/MPA.0b013e3181bba281

[43] Kubo K , Yamamoto K . IgG4‐related disease. Int. J. Rheum. Dis. 2016; 19(8): 747–62. 26259069 10.1111/1756-185X.12586

[44] Ito T , Nakamura T , Fujimori N *et al*. Characteristics of pancreatic diabetes in patients with autoimmune pancreatitis. J. Dig. Dis. 2011; 12(3): 210–6. 21615876 10.1111/j.1751-2980.2011.00498.x

[45] Khandelwal A , Inoue D , Takahashi N . Autoimmune pancreatitis: an update. Abdom. Radiol. (NY). 2020; 45(5): 1359–70. 31650376 10.1007/s00261-019-02275-x

[46] O'Reilly DA , Malde DJ , Duncan T *et al*. Review of the diagnosis, classification and management of autoimmune pancreatitis. World J. Gastrointest. Pathophysiol. 2014; 5(2): 71–81. 24891978 10.4291/wjgp.v5.i2.71 PMC4025075

[47] Sah RP , Chari ST , Pannala R *et al*. Differences in clinical profile and relapse rate of type 1 versus type 2 autoimmune pancreatitis. Gastroenterology. 2010; 139(1): 140–8; quiz e12–3. 20353791 10.1053/j.gastro.2010.03.054

[48] Sureka B , Rastogi A . Autoimmune pancreatitis. Pol J. Radiol. 2017; 82: 233–9. 28507644 10.12659/PJR.900899 PMC5413295

[49] Chari ST , Smyrk TC , Levy MJ *et al*. Diagnosis of autoimmune pancreatitis: the Mayo Clinic experience. Clin. Gastroenterol. Hepatol. 2006; 4(8): 1010–6 quiz 934. 16843735 10.1016/j.cgh.2006.05.017

[50] Zamboni G , Lüttges J , Capelli P *et al*. Histopathological features of diagnostic and clinical relevance in autoimmune pancreatitis: a study on 53 resection specimens and 9 biopsy specimens. Virchows Arch. 2004; 445(6): 552–63. 15517359 10.1007/s00428-004-1140-z

[51] Hirano K , Tada M , Isayama H *et al*. Long‐term prognosis of autoimmune pancreatitis with and without corticosteroid treatment. Gut. 2007; 56(12): 1719–24. 17525092 10.1136/gut.2006.115246 PMC2095691

[52] Manfredi R , Frulloni L , Mantovani W *et al*. Autoimmune pancreatitis: pancreatic and extrapancreatic MR imaging‐MR cholangiopancreatography findings at diagnosis, after steroid therapy, and at recurrence. Radiology. 2011; 260(2): 428–36. 21613442 10.1148/radiol.11101729

[53] Kamisawa T , Tu Y , Egawa N *et al*. Can MRCP replace ERCP for the diagnosis of autoimmune pancreatitis? Abdom. Imaging. 2009; 34(3): 381–4. 18437450 10.1007/s00261-008-9401-y

[54] Lee LK , Sahani DV . Autoimmune pancreatitis in the context of IgG4‐related disease: review of imaging findings. World J. Gastroenterol. 2014; 20(41): 15177–89. 25386067 10.3748/wjg.v20.i41.15177 PMC4223252

[55] Naitoh I , Nakazawa T , Okumura F *et al*. Endoscopic retrograde cholangiopancreatography‐related adverse events in patients with type 1 autoimmune pancreatitis. Pancreatology. 2016; 16(1): 78–82. 26626204 10.1016/j.pan.2015.10.011

[56] Sugumar A , Levy MJ , Kamisawa T *et al*. Endoscopic retrograde pancreatography criteria to diagnose autoimmune pancreatitis: an international multicentre study. Gut. 2011; 60(5): 666–70. 21131631 10.1136/gut.2010.207951

[57] Kamisawa T , Imai M , Yui Chen P *et al*. Strategy for differentiating autoimmune pancreatitis from pancreatic cancer. Pancreas. 2008; 37(3): e62–7. 18815540 10.1097/MPA.0b013e318175e3a0

[58] Takuma K , Kamisawa T , Tabata T *et al*. Utility of pancreatography for diagnosing autoimmune pancreatitis. World J. Gastroenterol. 2011; 17(18): 2332–7. 21633599 10.3748/wjg.v17.i18.2332 PMC3098401

[59] Horiuchi A , Kawa S , Hamano H *et al*. ERCP features in 27 patients with autoimmune pancreatitis. Gastrointest. Endosc. 2002; 55(4): 494–9. 11923760 10.1067/mge.2002.122653

[60] Iwasaki S , Kamisawa T , Koizumi S *et al*. Characteristic findings of endoscopic retrograde cholangiopancreatography in autoimmune pancreatitis. Gut Liver. 2015; 9(1): 113–7. 25167792 10.5009/gnl13473 PMC4282851

[61] Goodchild G , Pereira SP , Webster G . Immunoglobulin G4‐related sclerosing cholangitis. Korean J. Intern. Med. 2018; 33(5): 841–50. 30045615 10.3904/kjim.2018.018 PMC6129623

[62] Kim HJ , Kim YK , Jeong WK *et al*. Pancreatic duct “Icicle sign” on MRI for distinguishing autoimmune pancreatitis from pancreatic ductal adenocarcinoma in the proximal pancreas. Eur. Radiol. 2015; 25(6): 1551–60. 25501271 10.1007/s00330-014-3548-4

[63] Ufuk F . The “Duct‐penetrating sign” in inflammatory pancreatic mass. Abdom. Radiol. 2021; 46(3): 1280–2. 10.1007/s00261-020-02768-0 32940756

[64] Kalaitzakis E , Levy M , Kamisawa T *et al*. Endoscopic retrograde cholangiography does not reliably distinguish IgG4‐associated cholangitis from primary sclerosing cholangitis or cholangiocarcinoma. Clin. Gastroenterol. Hepatol. 2011; 9(9): 800–3.e2. 21699807 10.1016/j.cgh.2011.05.019 PMC3246637

[65] Farrell JJ , Garber J , Sahani D *et al*. EUS findings in patients with autoimmune pancreatitis. Gastrointest. Endosc. 2004; 60(6): 927–36. 15605008 10.1016/s0016-5107(04)02230-8

[66] Fujii‐Lau L , Levy MJ , Chari ST . Autoimmune pancreatitis. Atlas Endosc. Ultrasonogr. 2022: 113–6.

[67] Deshpande V , Mino‐Kenudson M , Brugge WR *et al*. Endoscopic ultrasound guided fine needle aspiration biopsy of autoimmune pancreatitis: diagnostic criteria and pitfalls. Am. J. Surg. Pathol. 2005; 29(11): 1464–71. 16224213 10.1097/01.pas.0000173656.49557.48

[68] Khandelwal A , Shanbhogue AK , Takahashi N *et al*. Recent advances in the diagnosis and management of autoimmune pancreatitis. AJR Am. J. Roentgenol. 2014; 202(5): 1007–21. 24758653 10.2214/AJR.13.11247

[69] Levy MJ , Reddy RP , Wiersema MJ *et al*. EUS‐guided trucut biopsy in establishing autoimmune pancreatitis as the cause of obstructive jaundice. Gastrointest. Endosc. 2005; 61(3): 467–72. 15758927 10.1016/s0016-5107(04)02802-0

[70] Kanno A , Masamune A , Fujishima F *et al*. Diagnosis of autoimmune pancreatitis by EUS‐guided FNA using a 22‐gauge needle: a prospective multicenter study. Gastrointest. Endosc. 2016; 84(5): 797–804.e1. 27068878 10.1016/j.gie.2016.03.1511

[71] Kurita A , Yasukawa S , Zen Y *et al*. Comparison of a 22‐gauge Franseen‐tip needle with a 20‐gauge forward‐bevel needle for the diagnosis of type 1 autoimmune pancreatitis: a prospective, randomized, controlled, multicenter study (COMPAS study). Gastrointest. Endosc. 2020; 91(2): 373–81.e2. 31654634 10.1016/j.gie.2019.10.012

[72] de Pretis N , Crinò SF , Frulloni L . The Role of EUS‐Guided FNA and FNB in autoimmune pancreatitis. Diagnostics (Basel). 2021; 11(9): 1653. 34573995 10.3390/diagnostics11091653 PMC8470670

[73] Moon SH , Kim MH , Park DH *et al*. IgG4 immunostaining of duodenal papillary biopsy specimens may be useful for supporting a diagnosis of autoimmune pancreatitis. Gastrointest. Endosc. 2010; 71(6): 960–6. 20304394 10.1016/j.gie.2009.12.004

[74] Moon SH , Kim MH . The role of endoscopy in the diagnosis of autoimmune pancreatitis. Gastrointest. Endosc. 2012; 76(3): 645–56. 22898422 10.1016/j.gie.2012.04.458

[75] Hamano H , Kawa S , Horiuchi A *et al*. High serum IgG4 concentrations in patients with sclerosing pancreatitis. N. Engl. J. Med. 2001; 344(10): 732–8. 11236777 10.1056/NEJM200103083441005

[76] Raina A , Krasinskas AM , Greer JB *et al*. Serum immunoglobulin G fraction 4 levels in pancreatic cancer: elevations not associated with autoimmune pancreatitis. Arch. Pathol. Lab. Med. 2008; 132(1): 48–53. 18181673 10.5858/2008-132-48-SIGFLI

[77] Rock B , Martins CR , Theofilopoulos AN *et al*. The pathogenic effect of IgG4 autoantibodies in endemic pemphigus foliaceus (Fogo selvagem). N. Engl. J. Med. 1989; 320(22): 1463–9. 2654636 10.1056/NEJM198906013202206

[78] Bhol K , Mohimen A , Ahmed AR . Correlation of subclasses of IgG with disease activity in pemphigus vulgaris. Dermatology. 1994; 189(Suppl 1): 85–9. 8049571 10.1159/000246938

[79] Aalberse RC , Van Milligen F , Tan KY *et al*. Allergen‐specific IgG4 in atopic disease. Allergy. 1993; 48(8): 559–69. 8116855 10.1111/j.1398-9995.1993.tb00749.x

[80] Ito T , Nakano I , Koyanagi S *et al*. Autoimmune pancreatitis as a new clinical entity. Three cases of autoimmune pancreatitis with effective steroid therapy. Dig. Dis. Sci. 1997; 42(7): 1458–68. 9246047 10.1023/a:1018862626221

[81] Kwon S , Kim MH , Choi EK . The diagnostic criteria for autoimmune chronic pancreatitis: it is time to make a consensus. Pancreas. 2007; 34(3): 279–86. 17414049 10.1097/MPA.0b013e31802eff5f

[82] Moon SH , Kim MH , Park DH *et al*. Is a 2‐week steroid trial after initial negative investigation for malignancy useful in differentiating autoimmune pancreatitis from pancreatic cancer? A prospective outcome study. Gut. 2008; 57(12): 1704–12. 18583399 10.1136/gut.2008.150979

[83] Ghazale A , Chari ST , Zhang L *et al*. Immunoglobulin G4‐associated cholangitis: clinical profile and response to therapy. Gastroenterology. 2008; 134(3): 706–15. 18222442 10.1053/j.gastro.2007.12.009

[84] Sandrasegaran K , Menias CO . Imaging in autoimmune pancreatitis and immunoglobulin G4‐related disease of the abdomen. Gastroenterol. Clin. North Am. 2018; 47(3): 603–19. 30115440 10.1016/j.gtc.2018.04.007

[85] Kallel L , Naija N , Boubaker J *et al*. La pancréatite auto immune: Revue systématique de la littérature [Autoimmune pancreatitis: A systematic review]. Tunis Med. 2011; 89(3): 221–30. 21387223

[86] Hart PA , Krishna SG , Okazaki K . Diagnosis and management of autoimmune pancreatitis. Curr. Treatment Options Gastroenterol. 2017; 15(4): 538–47. 10.1007/s11938-017-0147-x 28842855

[87] Okazaki K , Chari ST , Frulloni L *et al*. International consensus for the treatment of autoimmune pancreatitis. Pancreatology. 2017; 17(1): 1–6. 28027896 10.1016/j.pan.2016.12.003

[88] Wallace ZS , Mattoo H , Mahajan VS *et al*. Predictors of disease relapse in IgG4‐related disease following rituximab. Rheumatology. 2016; 55(6): 1000–8. 26888853 10.1093/rheumatology/kev438 PMC4900135

[89] Ito T , Nishimori I , Inoue N *et al*. Treatment for autoimmune pancreatitis: consensus on the treatment for patients with autoimmune pancreatitis in Japan. J. Gastroenterol. 2007; 42(Suppl 18): 50–8. 17520224 10.1007/s00535-007-2051-y

[90] Kamisawa T , Shimosegawa T , Okazaki K *et al*. Standard steroid treatment for autoimmune pancreatitis. Gut. 2009; 58(11): 1504–7. 19398440 10.1136/gut.2008.172908

[91] Masamune A , Nishimori I , Kikuta K *et al*. Randomised controlled trial of long‐term maintenance corticosteroid therapy in patients with autoimmune pancreatitis. Gut. 2017; 66(3): 487–94. 27543430 10.1136/gutjnl-2016-312049

[92] Hart PA , Topazian MD , Witzig TE *et al*. Treatment of relapsing autoimmune pancreatitis with immunomodulators and rituximab: the Mayo Clinic experience. Gut. 2013; 62(11): 1607–15. 22936672 10.1136/gutjnl-2012-302886

[93] Kim HM , Chung MJ , Chung JB . Remission and relapse of autoimmune pancreatitis: focusing on corticosteroid treatment. Pancreas. 2010; 39(5): 555–60. 20182397 10.1097/MPA.0b013e3181c8b4a5

[94] Hart PA , Kamisawa T , Brugge WR *et al*. Long‐term outcomes of autoimmune pancreatitis: a multicentre, international analysis. Gut. 2013; 62(12): 1771–6. 23232048 10.1136/gutjnl-2012-303617 PMC3862979

[95] Nagpal SJS , Sharma A , Chari ST . Autoimmune pancreatitis. Off. J. Am. College Gastroenterol. ACG. 2018; 113(9): 1301. 10.1038/s41395-018-0146-0 29910463

[96] Majumder S , Takahashi N , Chari ST . Autoimmune pancreatitis. Dig. Dis. Sci. 2017; 62(7): 1762–9. 28365915 10.1007/s10620-017-4541-y

[97] Carruthers MN , Topazian MD , Khosroshahi A *et al*. Rituximab for IgG4‐related disease: a prospective, open‐label trial. Ann. Rheum. Dis. 2015; 74(6): 1171–7. 25667206 10.1136/annrheumdis-2014-206605

[98] Soliman H , Vullierme MP , Maire F *et al*. Risk factors and treatment of relapses in autoimmune pancreatitis: rituximab is safe and effective. United Eur. Gastroenterol. J. 2019; 7(8): 1073–83. 10.1177/2050640619862459 PMC6794684 31662864

[99] Backhus J , Neumann C , Perkhofer L *et al*. A follow‐up study of a European IGG4‐related disease cohort treated with rituximab. J. Clin. Med. 2021; 10(6): 1329. 33807051 10.3390/jcm10061329 PMC8004657

[100] Asano J , Watanabe T , Oguchi T *et al*. Association between immunoglobulin G4‐related disease and malignancy within 12 years after diagnosis: an analysis after longterm followup. J. Rheumatol. 2015; 42(11): 2135–42. 26472416 10.3899/jrheum.150436

[101] Yamamoto M , Takahashi H , Tabeya T *et al*. Risk of malignancies in IgG4‐related disease. Mod. Rheumatol. 2012; 22(3): 414–8. 21894525 10.1007/s10165-011-0520-x

[102] Okamoto A , Watanabe T , Kamata K *et al*. Recent updates on the relationship between cancer and autoimmune pancreatitis. Intern. Med. 2019; 58(11): 1533–9. 30713326 10.2169/internalmedicine.2210-18 PMC6599917

[103] Shiokawa M , Kodama Y , Yoshimura K *et al*. Risk of cancer in patients with autoimmune pancreatitis. Off. J. Am. Coll. Gastroenterol. | ACG. 2013; 108(4): 610–7. 10.1038/ajg.2012.465 23318486

[104] Ikeura T , Miyoshi H , Uchida K *et al*. Relationship between autoimmune pancreatitis and pancreatic cancer: a single‐center experience. Pancreatology. 2014; 14(5): 373–9. 25278307 10.1016/j.pan.2014.04.029

